# The Benefit of Early Epidural Corticosteroid Injections for Acute Sciatica-Associated Lower Back Pain: A Four-Year Case Series in Lebanon

**DOI:** 10.7759/cureus.34847

**Published:** 2023-02-10

**Authors:** Ammar Chemeisani, Ali Hamade, Abed AlRaouf Kawtharani, Hasan Tarhini, Nour Hamze, Ali Msheik

**Affiliations:** 1 Neurological Surgery, Lebanese University Faculty of Medical Sciences, Hadath, LBN; 2 Internal Medicine, Lebanese University Faculty of Medical Sciences, Hadath, LBN; 3 Emergency Medicine, Lebanese University Faculty of Medical Sciences, Hadath, LBN; 4 General Practice, Lebanese University Faculty of Medical Sciences, Hadath, LBN; 5 Neurological Surgery, Zahraa Hospital University Medical Center (UMC), Beirut, LBN

**Keywords:** nsaids, nsaids, macnab criteria, nrs pain score, randomized retrospective study, sciatica, epidural steroid injections

## Abstract

Background

Early epidural steroid injections are currently widely used for patients experiencing lumbago. However, there is uncertainty about their efficacy, such as the limitation of continuous drug infusion and the need for well-trained physicians on this technique. The main objective of this study was to evaluate the effectiveness of early epidural steroid injections in treating patients with acute sciatica in the lower back in terms of symptom relief and recurrence rate.

Methods

A case series was conducted in Lebanon from 2015 to 2019. We recruited 98 patients suffering from sciatica due to disc disease over three-time intervals: two weeks, one, and three months. The immediate results accounted for the intensity of various symptoms (numerical rating scale (NRS) for pain) and the assessment of patient satisfaction (Macnab criteria).

Results

The clinical results showed at least a three-point pain relief according to Numerical Rating Scale (NRS) and a good grade according to MacNab (P <0.001), with only 10.4% of the total population having a positive leg raise test post-injection. The maximum benefit was noted after two weeks from the injection with a 5.7 mean change in NRS (p<0.001) with a good/excellent response in MacNab and a 4.9 change with only a good response after one month. This study noticed a rebound phenomenon where around half of the patients needed two steroid injections after three months (39.6 % after three months and 17.9 % after six months).

Conclusion

Even though current guidelines worldwide may suggest the use of conservative treatment for low back pain with acute sciatica, our study has demonstrated the effectiveness of epidural steroid injections in the Lebanese population with a significant outcome.

## Introduction

The latest studies done worldwide have shown that back pain has been placed in the top five diseases in terms of medical consultation, the reason for hospitalization, and surgical intervention. This led to high costs for the government, especially in terms of medical insurance and off-work days [1,2]. In most cases, back pain is due to a benign condition involving disc herniation with sciatic nerve compression. Back to pathophysiology, lumbosacral discs are mostly affected due to their weight-bearing capacity. A disc herniation will lead to the stretching of the surrounding tissues and compression of the surrounding structures (especially the sciatic nerve), which will induce an inflammatory cascade. Evidence has confirmed the presence of inflammatory cytokine release: Interleukin-6 (IL-6), IL-8, and prostaglandin E2 [3]. After the resulting inflammatory edema with direct neural compression, patients will experience low back pain and symptoms of nerve irritation, a phenomenon that is described as sciatica. Sciatica is the pain induced by the injured nerve over its dermatome [4]. We hypothesize that inflammation is predominant during the acute phase of sciatica and declines after several months in association with clinical improvement in most patients, so treating it in the early stages will relieve the pain. From this idea, epidural corticosteroids (steroids are potent anti-inflammatory agents) that are administered locally at the site of the intervertebral lesion are likely to be effective during the first weeks of an episode of sciatica [2].

Current guidelines worldwide suggest the use of conservative treatment (painkillers, oral vs. injections) as a first line. If it fails, invasive procedures such as epidural injections (EI) or surgical interventions are then required. Patients are non-compliant with treatment, especially in underdeveloped countries; whether pills pose grave side effects (GI irritation or kidney injuries from NSAIDS), daily commitment to the dose and their cost is significant. In addition, surgical treatment is often associated with complications such as local infections, iatrogenic neurovascular injury, or high morbidity rates (limited daily activities) [5]. Epidural steroid injections (ESI) offer a good solution; they consist of a single-dose administration of steroids via a needle under radiologic surveillance. It’s a minimally invasive procedure with a lower risk of infection than surgery [6]. In addition, it is a technique that offers an optimal delivery of the medicine to the affected area with almost no systemic effects in comparison to oral medications [7,8]. Thus, our main objective is to test the effectiveness of EI in patients suffering from acute sciatica. However, there is debate about their safety and effectiveness in terms of the limitation of continuous drug infusion and the need for well-trained physicians in this field regarding the fact that it’s a new technique. That’s why it is important to identify patients that benefit the most from those injections, and only a few trials have yet evaluated the effects in patients with acute sciatica [3]. Our study will focus on early EI for patients developing acute sciatica (defined as less than three months) after disc-associated back pain in the Lebanese population.

## Materials and methods

Study design

A case series was conducted from 2015 to 2019 in the Nabatieh area, South Lebanon, on 98 patients visiting the external clinics at Sheikh-Ragheb hospital having LBP with acute sciatica. The injection constituted 8 mg of dexamethasone and was administered under fluoroscopy guidance. The drug is delivered exactly in the area where the disc is compressing the nerve and causing inflammation when we injected steroids. The procedure is performed with the patient lying on their belly using fluoroscopic guidance, which helps to prevent damage to the nerve root. A radio-opaque dye is injected to enhance the fluoroscopic images and to confirm that the needle is properly placed. This technique allows the steroids to be placed closer to the irritated nerve root. The radiation exposure is minimal. The study was granted ethical committee clearance under the reference number: SRH2015-013. 

Subjects

From one hundred and six patients, 98 patients were selected according to the inclusion and exclusion criteria below (Table 1). 

**Table 1:** Inclusion and exclusion criteria

Inclusion criteria	Exclusion criteria
Patients with a positive diagnosis of disc bulging	Patients with cauda equina syndrome
Patients presenting with acute sciatica	Patients with ankylosing spondylitis
Failure of medical treatment for two weeks	Pregnant women
Age between 21 and 82 years	Patients with spinal tumors
	Patients with congenital musculoskeletal malformations

Data collection

First, to include the patients in our study, we confirmed the diagnosis of degenerative disc disease by the positive history of low back pain with confirmed magnetic imaging resonance (MRI) findings. We recorded the type of pain, irradiation, paresthesia, the location of the disc herniation, and the side of sciatica from the patient’s medical records. Our records also included the failure to respond to medical treatment taken over two weeks’ duration. The interrogation was done using the phone number obtained from the patient’s file, depending on the Numeric Rating Scale (NRS) and MacNab criteria [8]. A scale above six and a good or excellent criterion were indicators of the trial’s success. Visual Analogue Scale (VAS) score is a self-assessment tool for pain during which patients visually rate their pain level, giving it a number ranging between 0 (no pain at all) and 10 (the most intense pain that could ever be felt) [9,10]. MacNab criteria include four options (excellent, good, fair, or poor) to rate patients’ satisfaction with the surgical procedure [11].

The variables included in the datasheet collection were: age, gender, sciatica, radiculopathy, numbness, paresthesia, disc location, NRS score before injection, after two weeks, one month, and three months, leg raise test response before and after the injection, MacNab criteria after two weeks, one month, and three months, the necessity of another injection after two months, three months, or six months, and the necessity for the performance of surgery after nine months.

Statistical analysis

We used Microsoft Excel (Redmond, USA) for data entry and the tables and figures used as study results. We used an IBM Corp. Released 2016. IBM SPSS Statistics for Windows, Version 24.0. Armonk, NY: IBM Corp. for data analysis and statistical tests. A descriptive analysis was first done to assess the distribution of our sample. Means, standard deviations, and percentiles of continuous variables were expressed, and frequencies and percentages of categorical ones were calculated. Then, the repeated measures ANOVA test was used to determine the MacNab and NRS differences over time. The significance level of the test was admitted as p-value < 0.05. Repeated measures ANOVA is the equivalent of the one-way ANOVA used for related samples when it is intended to investigate the change in the mean over time, with at least three repeated measures at different time intervals. Repeated measures ANOVA output shows the within-subject effect firstly, showing whether there is a significant difference overall between the means at different time intervals. Second, it shows the pairwise comparisons, which allow discovering which specific means differed over time, so it shows the Bonferroni post hoc test. Finally, it shows the profile plot to show the trend of mean variation graphically [12].

## Results

Sociodemographic information

**Figure 1:**
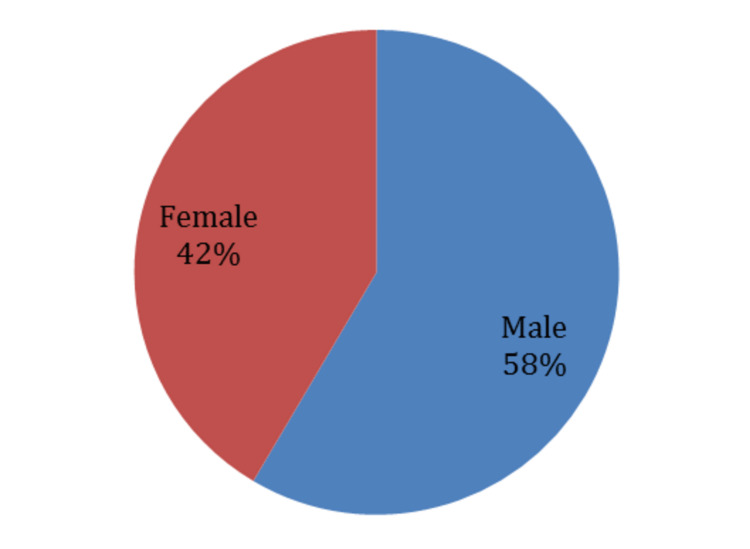
Gender distribution of the study participants

Eight patients used injection after two months: those people were not taken into consideration in analyzing scores variations because this will affect steroid injection assessment over three months. Otherwise, they will be introduced in our descriptive statistics. Our study participants had a mean age of 50 years (50.06 ± 15.604), ranging between 21 and 82 years. Due to their structural weakness, less developed muscle mass, and their dependence on spinal strength, especially in pregnancy, females are usually more prone to develop back pain due to disc herniation; 42% of our participants were females (Figure 1).

Clinical symptoms and signs

**Figure 2:**
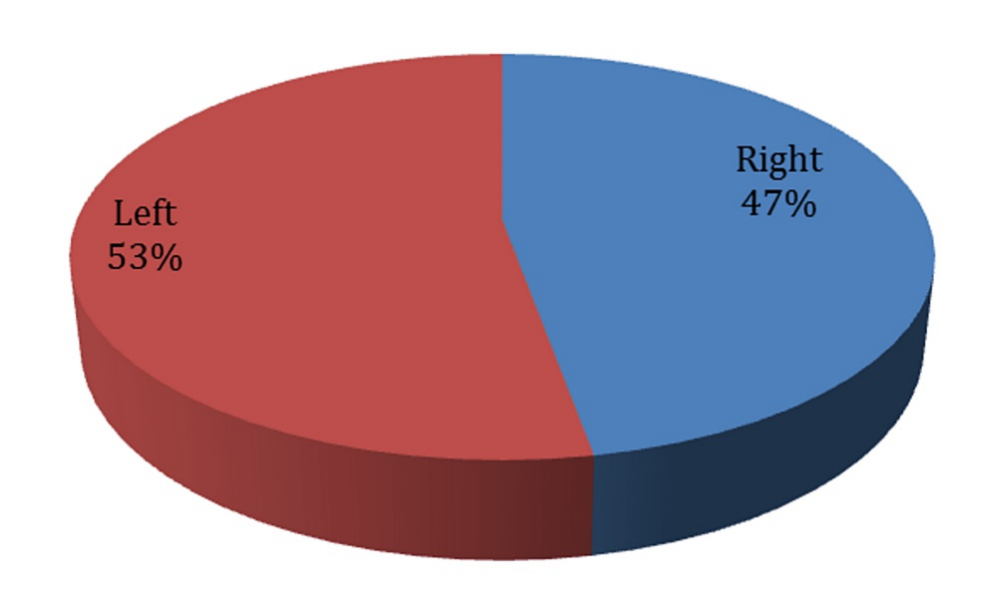
Sciatica symptom in patients

**Figure 3:**
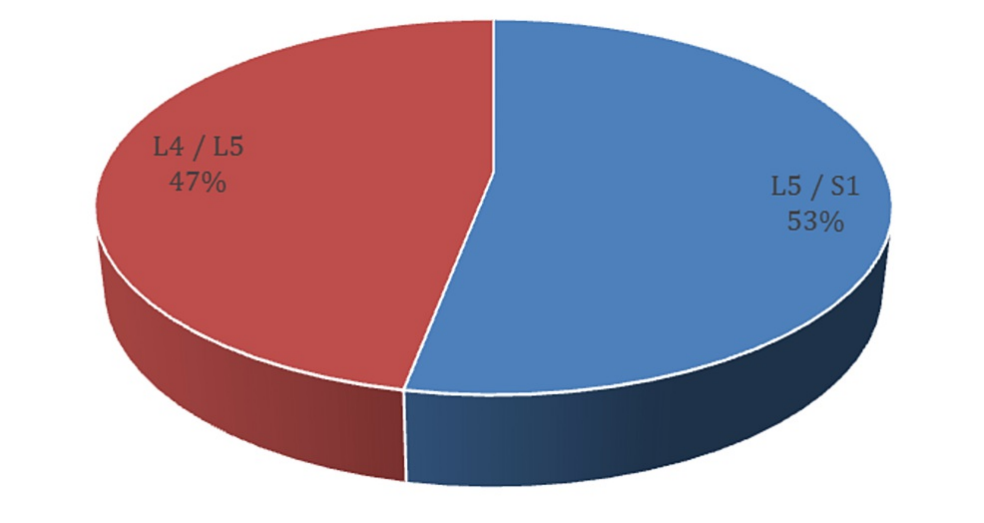
Radiculopathy in study participants

**Figure 4:**
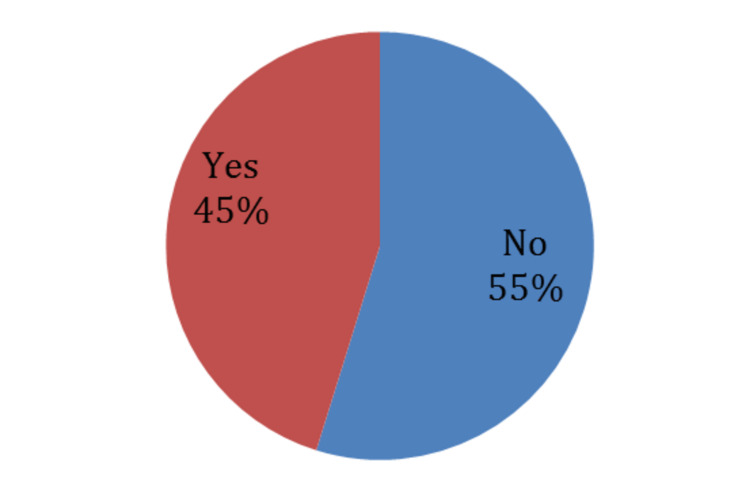
Numbness as a clinical symptom in study participants

**Figure 5:**
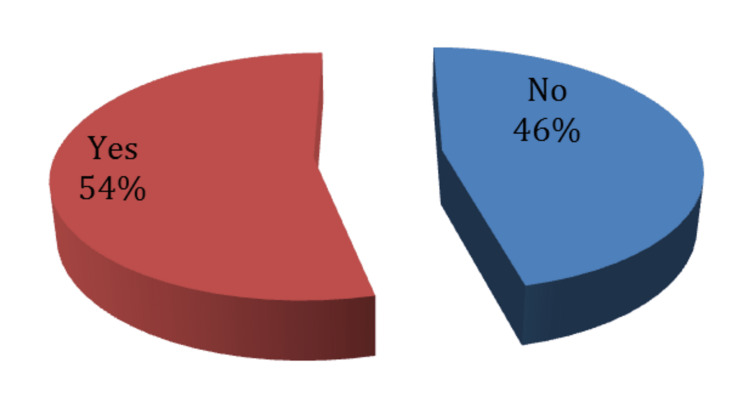
Paresthesia symptoms in the study participants

As for clinical symptoms, almost half of the patients (53%) had left-sided sciatica. Concerning radiculopathy, most of the patients (53%) had an L5/S1 radiculopathy, then comes the L4/L5 radiculopathy with 47% (Figure 2). This is expected since those discs are the most used, support heavy weights, and tolerate high pressures in spinal cord movements. As for numbness, almost half of our participants (45%) had numbness (Figures 3, 4, 5).

Almost half of our patients (54%) had paresthesia (Figure 5). Neurological compromises such as numbness and paresthesia are common findings since sciatica is related to nerve compression. However, in the early stages of sciatica, the patients can express only back pain irradiating along the sciatic nerve: numbness and pares; these are usually advanced findings related to late nerve suffering.

**Figure 6:**
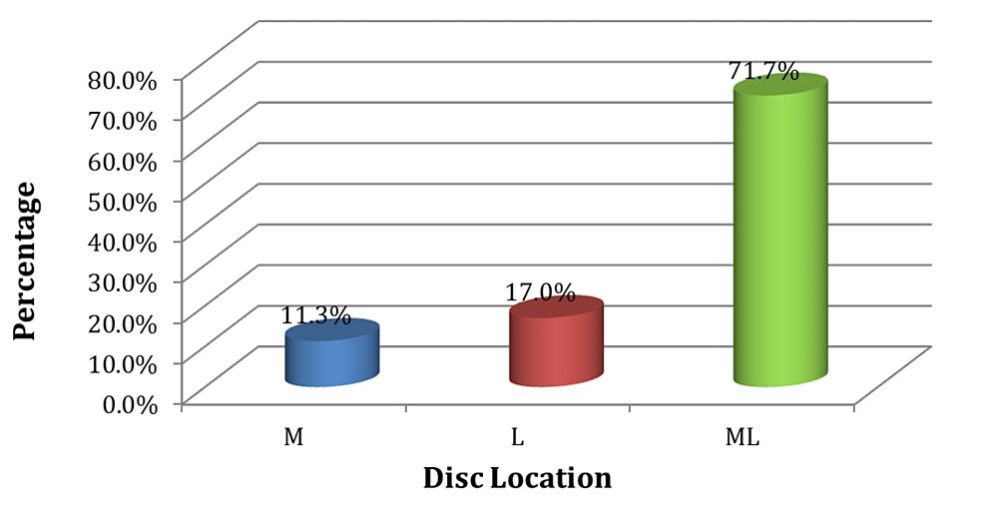
Disc location of the study participants Legends: M: medial; L: lateral; ML: medial and lateral

The majority of the participants (71.7%) had an “ML” (mediolateral) disc location (Figure 6). Back to the anatomy, the anterior and the posterior parts are relatively protected by the vertebral column and the layers of ligaments, respectively. Whereas the mediolateral part is a relative “escape “area for early disc herniation leading to a direct sciatic nerve compression: this table emphasized this point since the majority of the patients' mediolateral disc herniation (71.7%), whereas the others are less (11.3 for median and 17.3 for lateral which is an advanced stage).

**Figure 7:**
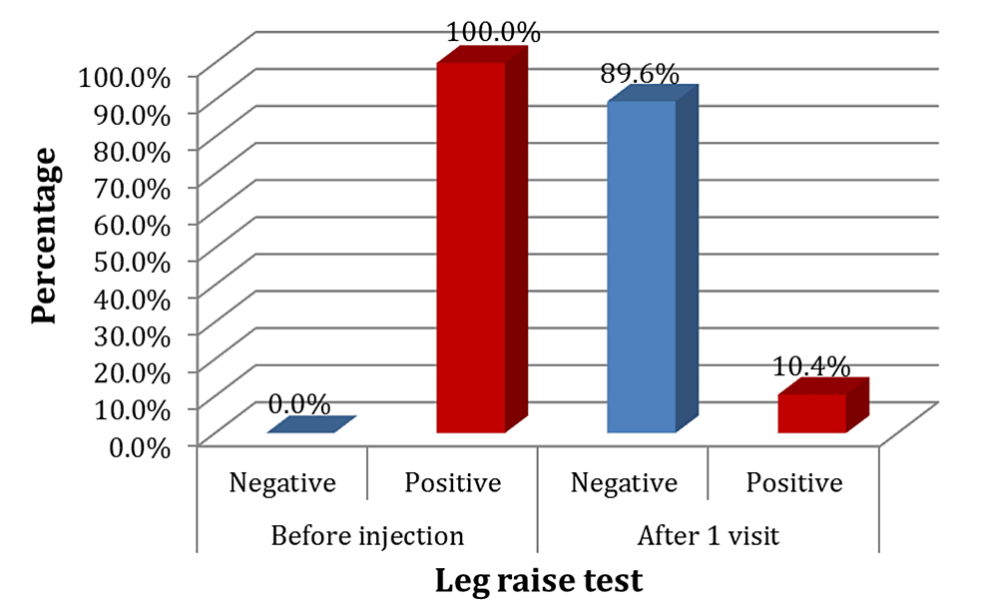
Leg raise associated pain before and after the injection of study participants

As for the leg raise test, while all the patients had a positive leg raise before injection, only 10.4% of them had a positive one after one visit (Figure 7).

Evolution

**Figure 8:**
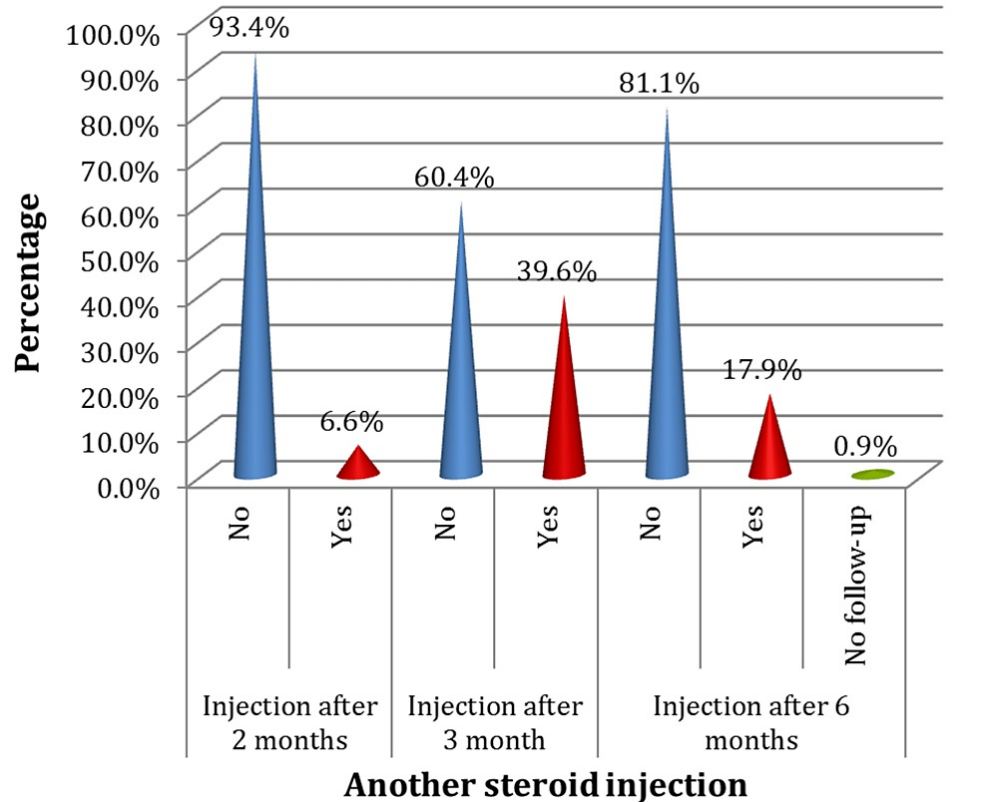
Distribution of the participants according to the need for another steroid injection at different time intervals

**Figure 9:**
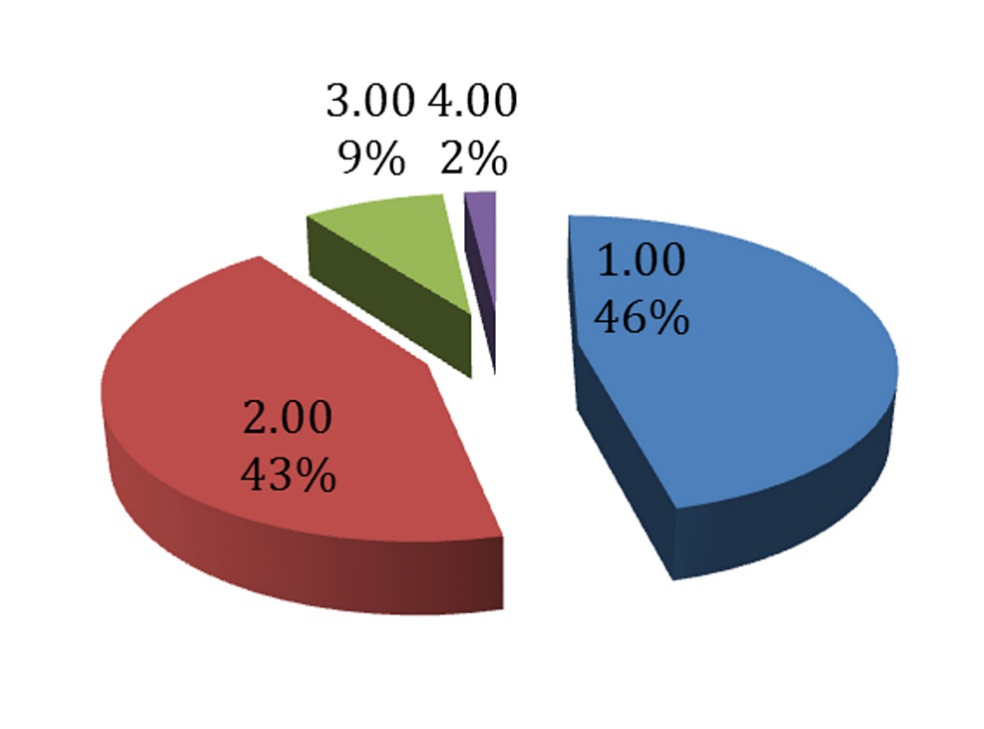
Distribution of participants according to the number of injections received

**Figure 10:**
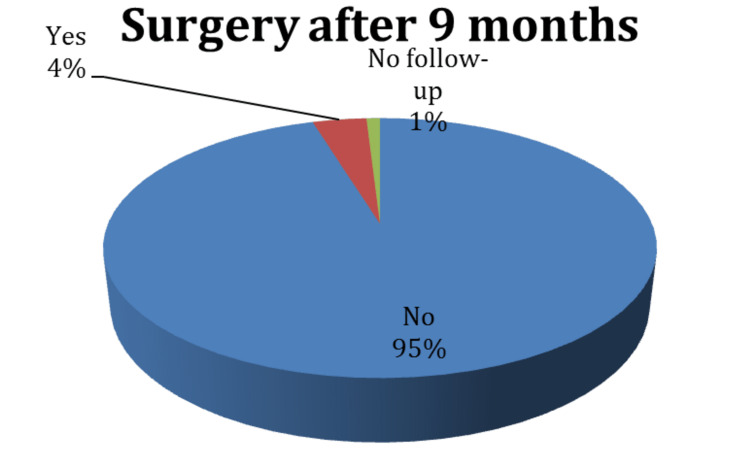
Post-injection surgery rate after nine months

The following figure reveals the need for another injection: only 6.6% of the patients needed another injection after two months, while 39.6% needed another injection after three months, and 17.9% needed one after six months. As for the number of injections needed, almost half of the trial participants (46%) had only one injection, 43% had two injections, and 9% of only 2% of the patients undertook four injections. Only 4% of the study participants needed surgery nine months following the injections, whereas almost all patients who underwent the injections did not require it (Figures 8, 9, 10).

Score variations

NRS

Using repeated measures ANOVA, Table 2 shows the mean NRS of the study participants before injection (7.22), after two weeks (1.95), after one month (2.43), and following three months (4.00). The score seems to get lower as time passes by. This observed overall difference is statistically significant (p < 0.001).

**Table 2:** Descriptive statistics (VAS score)

Variable	Mean	Std. Deviation	N	F	P- value (overall difference)
NRS before injection	7.22	1.206	98	F (2.23; 216.25) = 545.31	< 0.001
NRS after 2 weeks visit	1.95	1.992	98		
NRS after 1 month	2.43	2.215	98		
NRS after 3 months	4.00	2.154	98		

**Figure 11:**
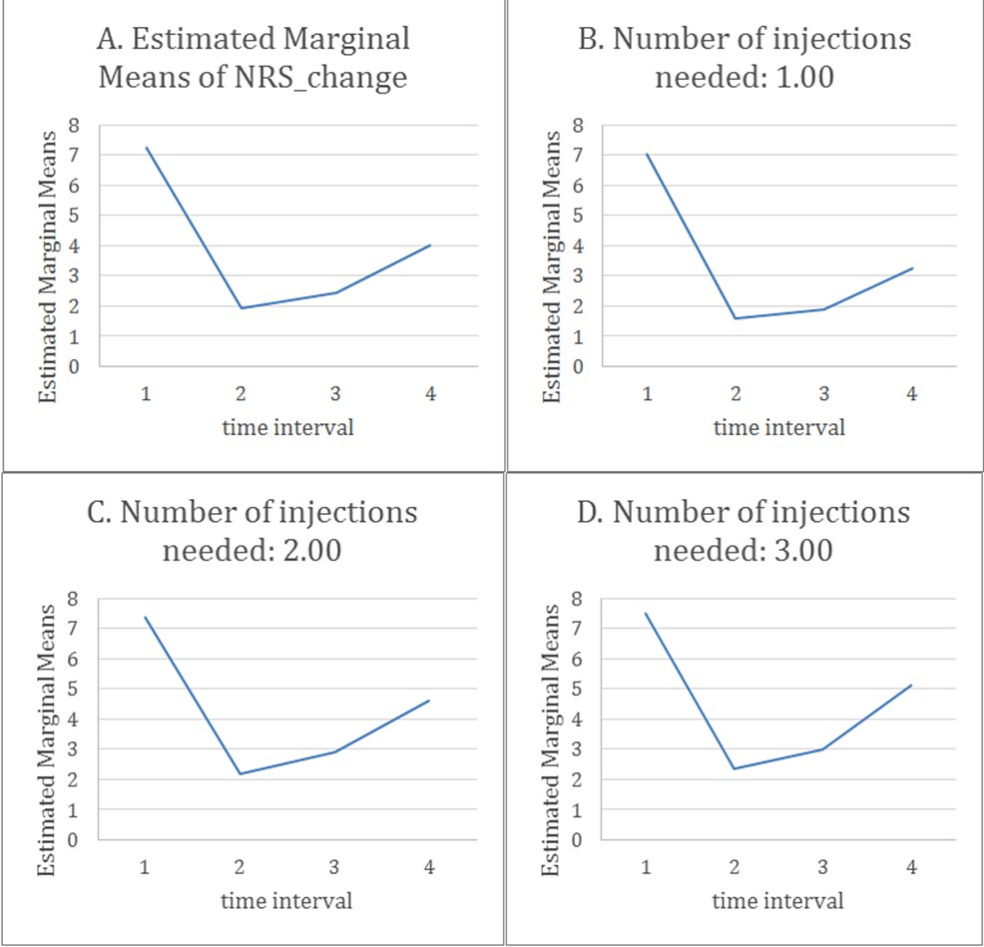
The mean change in NRS along the four-time interval (one before injection, two after two weeks, three after one month, and four after three months) by repeated measures ANOVA.

Four groups are formulated based on the number of injections to see if the latter affected the patients’ response: group A for all the patients studied, group B for those who received one injection, C for two injections, and D for three injections). In Figure 11A, the estimated marginal means of the NRS change is illustrated (all injections included), and its diminution is noted with a slight rebound effect at months three and four. We can notice the same shape of the curve in Figure 11B (where patients received only one injection), Figure 11C (two injections received), and Figure 11D (three injections).

Furthermore, pairwise comparisons among all the intervals using repeated measures ANOVA are depicted in Table 3. It demonstrates that the mean difference in NRS is statistically significant in different pairwise in all groups (p < 0.001). As mentioned above, NRS has maximal improvement by 5.2 points between the first and the second time interval. This improvement has been stabilized and slightly reduced by a 0.5 point between time 2 and time 3 with a p <0.001. Whereas after three months, the mean of NRS was 4 but never returned to baseline, making the difference around 3.2 points between time 1 and time 4: the results were statistically significant P<0.001 (Table 3).

**Table 3:** Pairwise comparisons (NRS)

(I) NRS		Mean Difference (I-J)	Std. Error	p-value	95% Confidence Interval for Difference	
					Lower Bound	Upper Bound
NRS score before the injection	NRS after 2 weeks	5.276^*^	0.155	< 0.001	4.859	5.692
	NRS after 1 month	4.796^*^	0.168	< 0.001	4.344	5.248
	NRS after 3 months	3.224^*^	0.172	< 0.001	2.761	3.688
NRS after 2 weeks	NRS before the injection	-5.276^*^	0.155	< 0.001	-5.692	-4.859
	NRS after 1 month	-.480^*^	0.073	< 0.001	-0.676	-0.283
	NRS after 3 months	-2.051^*^	0.145	< 0.001	-2.442	-1.660
NRS after 1 month	NRS before the injection	-4.796^*^	0.168	< 0.001	-5.248	-4.344
	NRS after 2 weeks	.480^*^	0.073	< 0.001	0.283	0.676
	NRS after 3 months	-1.571^*^	0.130	< 0.001	-1.921	-1.222
NRS after 3 months	NRS before injection	-3.224^*^	0.172	< 0.001	-3.688	-2.761
	NRS after 2 weeks	2.051^*^	0.145	< 0.001	1.660	2.442
	NRS after 1 month	1.571^*^	0.130	< 0.001	1.222	1.921

MacNab Criteria

MacNab criteria is not a scoring system by numbers: it consists of subjective information given by the patient and attributed to four parameters. For statistical analysis, every parameter was associated with a number where poor signifies “1”, fair signifies “2”, good signifies “3,” and excellent signifies “4”. All the patients were assumed to have a poor MacNab score before injections.

The MacNab score is overall significantly different through time from two weeks after the first injection to the following three months (p < 0.001) using repeated measures ANOVA. The maximal score was noticed after two weeks when the mean was 3.3 (good to excellent). As NRS, it has been stabilized to 3.05 (good-excellent) on time 3 with a p-value of 0.001; to return to 2.3 (fair-good) at the end of the study but never came to 1 and kept statistically significant (Table 4).

**Table 4:** Descriptive Statistics (MacNab)

Variable	Mean	Std. Deviation	N	p-value (overall difference)
MacNab after 2 weeks	3.30	0.819	98	< 0.001
MacNab after 1 month	3.05	0.953	98	
MacNab after 3 months	2.3861	0.96923	98	

**Figure 12:**
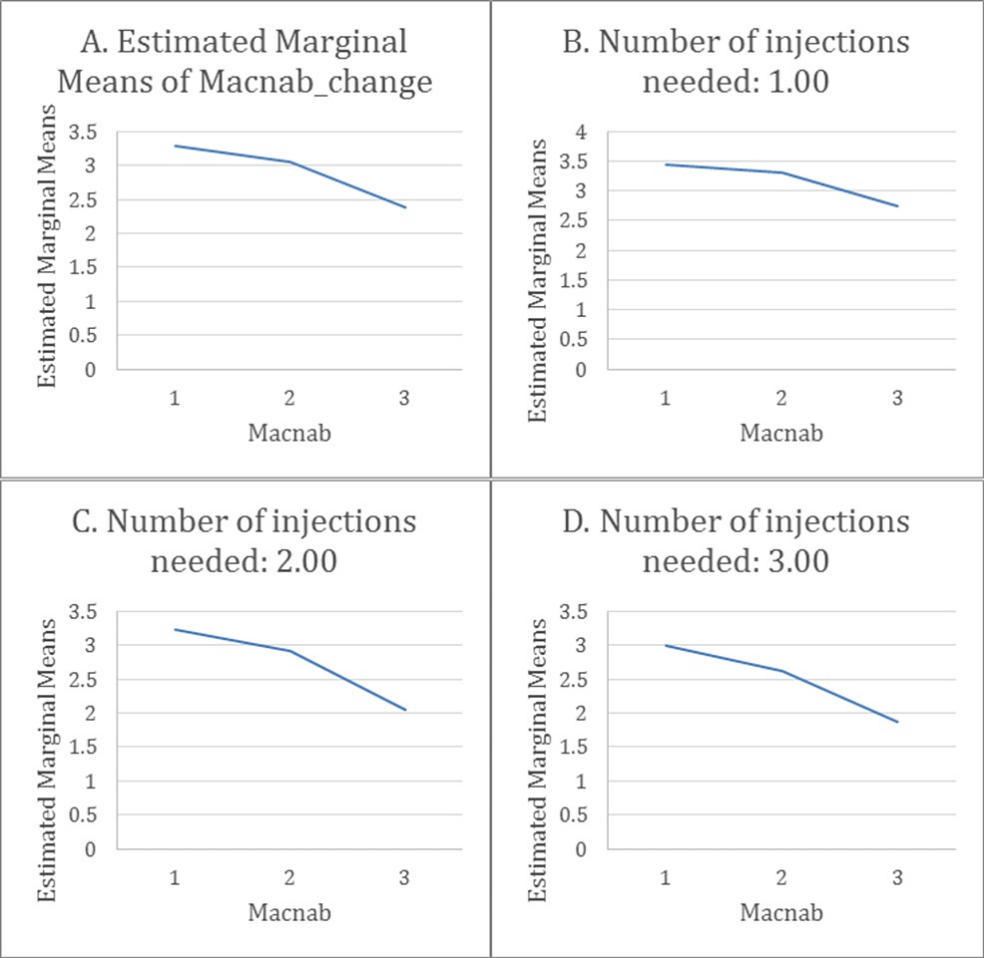
MacNab criteria change after the first injection

MacNab score reduction is valid regardless of the number of injections performed (Figure 12). Part A shows that Macnab is decreasing overall in mean (all injection numbers included). This decrease is also noted in patients who received one injection (Figure 12B), two injections (part C), or three injections (Figure 12D).

The mean difference in MacNab score is statistically significantly different pairwise in all the groups (p < 0.001). We can notice that during the study, the Macnab mean between two weeks and one month only decreased by 0.3, reflecting that the patients had significantly persistent improvement for around one month. This means only decreased by 1 point after three months. Even though the change was a slight decrease in improvement by 0.6 points between 1 month and 3 months, suggesting that the effect of the injection was almost stabilized in the same stage (Table 5).

**Table 5:** Pairwise Comparisons (MacNab score)

MacNab		Mean Difference (I-J)	Std. Error	P value	95% Confidence Interval for Difference	
					Lower Bound	Upper Bound
MacNab after 2 weeks	MacNab after 1 month	.248^*^	0.045	< 0.001	0.137	0.358
	Macnab after 3 months	.911^*^	0.069	< 0.001	0.743	1.079
MacNab after 1 month	MacNab after 2 weeks	-.248^*^	0.045	< 0.001	-0.358	-0.137
	MacNab after 3 months	.663^*^	0.060	< 0.001	0.517	0.810
MacNab after 3 months	MacNab after 2 weeks	-.911^*^	0.069	< 0.001	-1.079	-0.743
	MacNab after 1 month	-.663^*^	0.060	< 0.001	-0.810	-0.517

All in all, a three to four points change with a value of p<0.001 (Table 4) in NRS and a good response according to MacNab score (Table 5), with only 10.4% of the total population having a positive leg raise test post-injection are concluded. The maximum benefit was noted after two weeks from the injection with a 5.7 mean change in NRS (p<0.001) with a good/excellent response in MacNab and a 4.9 change with only a good response after one month. The results were still significant between the initiation of the study and the end: numbers never returned to baseline. The rebound phenomenon was noticed in this study, where around half of the patients needed two steroid injections after three months (39.6 % after three months and 17.9 % after six months).

## Discussion

The majority of the patients had L5-S1 disc disease, which was expected since this disc is one of the most weight-bearing spinal discs. Our study has shown that EI is an effective option in relieving acute sciatica due to degenerative disc disease over six months, shown by a significant change in NRS; and a good response according to MacNab score, as well as a low rate of positive leg raise test post-injection. This finding matches many other studies, where in 2009, a review found EI steroid injection fairly effective but for short-term pain relief [13]. Our results suggest that EI can be an effective option for a minimum of three months period. This joins another research team who found that 75.8% of their patients had an 80% pain reduction after three months of EI injections repeated once to five times [14].

This efficacy of EI is translated in terms of symptom relief (3 to 4 points improvement), cost (only a single injection), and side effects that can appear after using over-the-counter medications for a long period. Mac Vigar and his colleagues proved in their systematic review that for pain originating from lumbar disc herniation, more than half the patients (60%) had at least 50% less pain during the first two months after the injection, but pain relief was maintained for one year in only 40% of the patients [15]. In our study, the maximum benefit was noted after two weeks from the injection. After three months, we notice that the patient’s pain score was still low, suggesting that the injection effect lasted to this period but did not return to the pre-injection baseline.

There was no significant difference between the people who needed one, two, or even three injections. The overall changes in MacNab and NRS are the same; thus, any patient presenting with lower back pain who undergoes a steroid spinal injection is supposed to have one of these outcomes: either a symptom-free interval for six months or he/she might need another injection after that. To our knowledge, the literature doesn’t offer much about the relationship between the number of injections and symptom relief. Therefore, this study adds good value to the scientific community by pointing out that patients' symptom relief doesn’t rely mainly on the higher number of injections but rather on the appropriate technique and correct indication applied at the suitable setting for the patients. This also suggests that the cost of EI isn’t expected to be high, especially since some patients needed only one injection and got great results that were able to even delay or cancel their need for surgical intervention. As mentioned earlier, the appearance of the symptoms after several months (described as a rebound phenomenon) was documented in our study, where the patients seemed to have a slightly higher NRS after three months, but it was still significantly much lower than the baseline [2]. In addition, around 54% of patients needed one or more injections after three months.

Our results showed significant improvement in patients’ symptoms and less need for surgery when almost all patients postponed the surgery option, and only 4% required to be operated on. This joins the findings of Bartelson et al., who argued that EI must allow patients to avoid surgical intervention [16]. Besides, a randomized trial had proved EI to be effective in avoiding surgical intervention [1]. However, the North American Spine Society doesn’t recommend EI in the setting of surgery delay alone [17]. The clinically significant results, the maximum response in the early phase post-injection, the diminished response between first and second injections, in addition to the reappearance of symptoms after “the supposed time of inflammatory waning” are all evidence supporting that early intervention can target early inflammation.

Study strengths

The size of our population is of value, and the patients were all diagnosed based on highly sensitive imaging findings (MRI), so no false-positive diagnoses were present. All of the participants presented to our neurosurgeon clinic for re-evaluation. During the study phase, the patients mentioned following the strict recommendations of the doctor and did not take any other medical advice. Moreover, our surgeon has done over five hundred epidural injections during his career with minimal complications classifying him as an expert in the field. Besides, we used two scoring systems for patients’ evaluation.

Study limitations

The need for single or double injections is beyond the scope of this study due to the lack of further parameters to be taken into consideration (vascular compromise, immune compromise, work pattern, physiotherapy sessions, comorbidities, etc.). One of the limitations was the assessment of patients after one month, which was performed over the phone and not on a clinical basis; thereby, accurate data were not obtained. Medical insurance did not cover the cost of injections, so some patients would not reveal truly if they improved or not. Our study only covered six months and did not follow the patients, further especially that the rebound phenomenon after more than six months was mentioned in the literature. The absence of a control group was another limitation since the majority of the patients have taken analgesics earlier with no improvement or had directly chosen the injection option.

Study perspectives

After proving the benefit of EI in Lebanese patients, future studies need to have a prospective design with a follow-up of at least one year to reach the long-term benefits of EI. Besides, the delay or cancelation of surgeries should be tackled hugely with a better assessment and understanding of the settings during which patients rely on EI and avoid surgical procedures. Moreover, larger study samples are needed to have solid recommendations that enable lumbar disc herniation patients to have better follow-up and intervention. Finally, it might be interesting to evaluate the difference in the quality of life, functional activity, and cost in the patient’s daily life having two groups: EI and surgery patients.

## Conclusions

Our study has shown that EI is an effective option in relieving acute sciatica due to degenerative disc disease over six months. We witnessed a significant change in NRS and a good response according to MacNab's score. Besides, the maximum benefit was noted after two weeks from the injection, but a rebound phenomenon was noticed in half of the patients who needed two steroid injections after three months. This supports that early intervention can target early inflammation. Our results also suggested that EI can be an effective option for a minimum of three months period. This efficacy is translated in terms of symptom relief, cost (only a single injection in the majority of the cases), and side effects of long periods of using over-the-counter medications. However, the need for a single or a second injection is beyond the scope of this study, but the excellent response at two weeks and one month can have a link with the need for a single injection. In conclusion, steroid injections in acute sciatica are a very effective and safe procedure to be adopted. The response rate was adequate for the price and the duration effect of these injections. It reduces the pain severity, improves the quality of life, and reduces/postpones the surgery. Steroid site infection was very minimal. This treatment is a pain reliever, not a disease controller, and the patient might re-express the symptoms if he does not start some rehabilitation therapy. Further studies are needed worldwide to emphasize its effectiveness.
